# Disease probability-enhanced follow-up chest X-ray radiology report summary generation

**DOI:** 10.1038/s41598-025-12684-2

**Published:** 2025-07-24

**Authors:** Zhichuan Wang, Qiao Deng, Tiffany Y. So, Wan Hang Chiu, Kinhei Lee, Edward S. Hui

**Affiliations:** 1 Department of Imaging and Interventional Radiology, The Chinese University of Hong Kong, HKSAR, China; 2 The CU Lab for AI in Radiology (CLAIR), The Chinese University of Hong Kong, HKSAR, China; 3 Hospital Authority, HKSAR, China; 4 Department of Psychiatry, The Chinese University of Hong Kong, HKSAR, China; 5 Gerald Choa Neuroscience Institute, The Chinese University of Hong Kong, HKSAR, China

**Keywords:** Biomedical engineering, Computer science

## Abstract

A chest X-ray radiology report describes abnormal findings not only from X-ray obtained at a given examination, but also findings on disease progression or change in device placement with reference to the X-ray from previous examination. Majority of the efforts on automatic generation of radiology report pertain to reporting the former, but not the latter, type of findings. To the best of the authors’ knowledge, there is only one work dedicated to generating summary of the latter findings, i.e., follow-up radiology report summary. In this study, we propose a transformer-based framework to tackle this task. Motivated by our observations on the significance of medical lexicon on the fidelity of report summary generation, we introduce two mechanisms to bestow clinical insight to our model, namely disease probability soft guidance and masked entity modeling loss. The former mechanism employs a pretrained abnormality classifier to guide the presence level of specific abnormalities, while the latter directs the model’s attention toward medical lexicon. Extensive experiments were conducted to demonstrate that the performance of our model exceeded the state-of-the-art.

## Introduction

Chest X-ray radiology report generation model can automatically report radiological findings on radiogram in an end-to-end manner^1–6^, potentially providing a means to alleviate the workloads of radiologists. A major shortcoming of the majority of these models is that they are limited to reporting findings that are present in a single chest X-ray examination. However radiologist typically writes report based not only on a single (follow-up) X-ray but also on the X-ray from previous check-up to discern change in disease severity, and/or change in the placement of medical device. This type of radiology report is denoted as *follow-up radiology report*. In other words, majority of the current report generation models fail to report these disease progression-related findings, and are thus not suitable for the follow-up radiology report generation task.

**Fig. 1:**
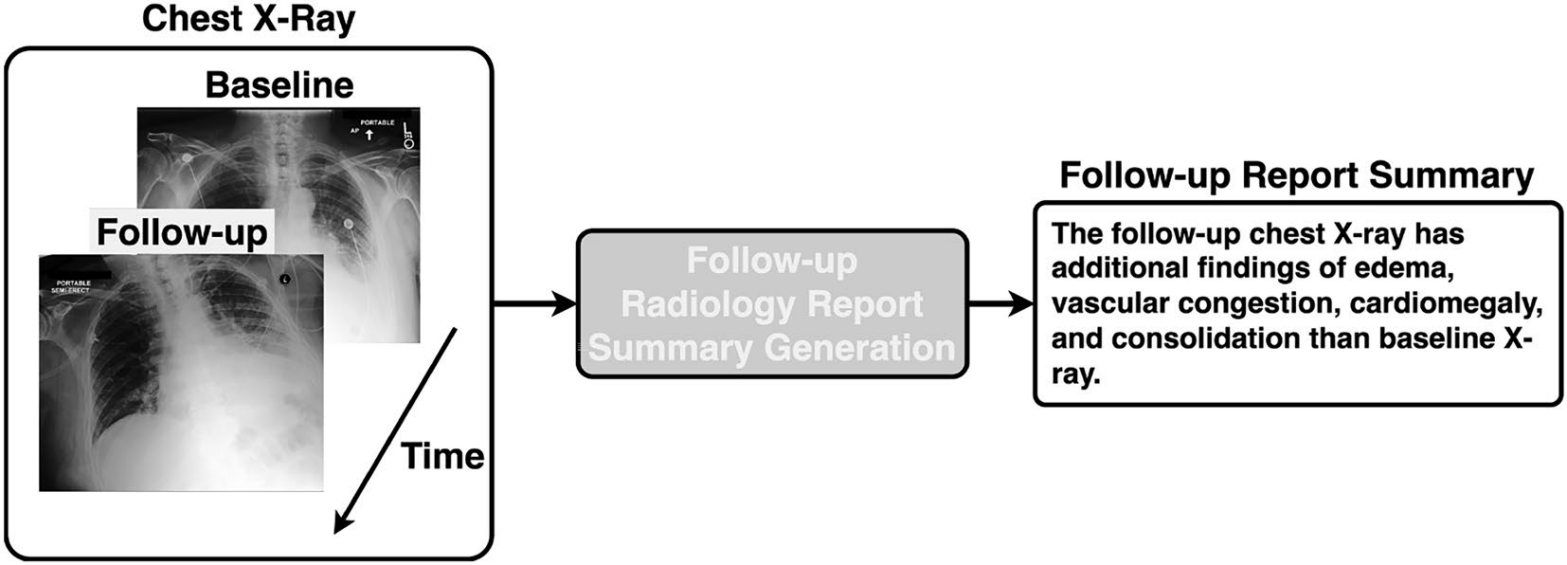
A schematic of our proposed model that generates a textual summary of disease progression from a pair of chest X-rays taken at follow-up and baseline examinations.

Considering that automatic generation of a follow-up radiology report is a clinically relevant and important problem, this study aims to develop an end-to-end model to tackle a related and simplified generative problem, dubbed follow-up radiology report summary generation, whereby a textual summary of disease progression is generated based on a pair of chest X-rays obtained at follow-up and baseline examinations (see Fig. 1). There are two key challenges to the generation of follow-up report summary. The first is the diagnostic accuracy of the generated report summary. Take a ground truth follow-up report summary – *The follow-up chest X-ray is missing the finding of pneumonia compared to the baseline X-ray* – as an example. It is acceptable if the model mis-predicts the word *“of”*, but not acceptable when the model makes the wrong diagnosis of the abnormality – *pneumonia*. It is therefore of paramount importance to maintain the clinical accuracy of generated reports. The other issue is related to the suboptimal use of equal attention to each word in the generated report under the supervision of the commonly used masked language modeling (MLM) loss, e.g.,^1,2,7^. As previously mentioned, the clinical accuracy of a generated report summary is more important than the fidelity of other generated words. Abnormalities-related entities should therefore deserve more attention than other words.

In this study, we therefore propose two mechanisms to partly address each of these two issues, whereby additional diagnostic information is provided, and model attention is reallocated in the hope of improving the prediction of medical lexicon. These mechanisms, respectively dubbed *disease probability soft guidance* and *masked entity modeling loss*, aim to bestow clinical insight on the follow-up report summary generation task. Disease probability soft guidance employs a pretrained abnormality classifier to provide complementary guidance on the presence level of abnormalities commonly seen on chest X-ray, whilst masked entity modeling loss directs the model’s attention toward abnormality-related words. In this study, our proposed framework was tested on two groups of follow-up findings, namely change in the presence or absence of abnormalities and change in the severity of abnormalities (see Table 1 for the full list of follow-up findings being investigated). A preliminary version of this work has been reported in^8^. Our contributions are:

- A framework was proposed to tackle the follow-up chest X-ray radiology report summary generation task.
- Two mechanisms, namely disease probability soft guidance and masked entity modeling loss, were proposed to bestow clinical insight on disease-related words.
- Comprehensive experiments were conducted to demonstrate that the performance of our model exceeded the state-of-the-arts.

**Table 1:** Enumerations of all possible follow-up findings on which our proposed framework, disease probability-enhanced (DPE) follow-up chest X-ray radiology report summary generation, were tested.

Type of follow-up finding	Example
Absence of previous finding	The follow-up image is missing the findings of pleural effusion and atelectasis than the baseline image.
Presence of new finding	The follow-up image has an additional finding of pneumothorax than the baseline image.
Change in disease severity	The level of atelectasis has changed from minimal to mild.
No change in disease status	Nothing has changed.

## Related work

### Report generation from a single chest X-ray

Chest X-ray radiology report generation models were first based on CNN/RNN architecture^5,6^, whereby CNN and RNN respectively served as image encoder and text decoder. Later, with the advent of transformer that excels in generative tasks^9^, the performance of report generation model improved after adopting the transformer architecture, e.g.,^1,2,4,10,11^. Miura et al. proposed a transformer-based encoder-decoder architecture together with two losses to optimize the completeness and consistency of the generated reports^2^. Yan et al. proposed a visual prior-guided transformer framework, whereby the prior was extracted by performing unsupervised clustering and sparse attention on image patches^11^. To further improve the semantic consistency of the generated reports, Tao et al. proposed a memory bank of disease-related multimodal representations for fine-grained feature consolidation, and the embedding of which was utilized in the report decoding process^4^. Tanida et al. combined transformer-based report generator with an anatomy detector for generating report that was conditioned on the user-defined bounding box in the hope of further improving model fidelity^1^.

### Longitudinal representation

X-ray is the most common imaging modality for the diagnosis of cardiothoracic conditions, and the progression of the thereof. Radiologists often write reports based not only on the current (follow-up) X-ray, but also on that from previous examination and the corresponding report. In view of this, Bannur et al. have proposed a vision-language pretraining framework with two identical visual encoders that took the X-ray from baseline and follow-up examinations of the same patient for generating the radiology report and classification of disease progression for the follow-up X-ray^12^. Serra et al. proposed a framework that extracted visual anatomical features from a pair of chest X-ray using a single faster R-CNN, which were subsequently aligned, concatenated and projected onto a joint representation for generating report sentences that corresponded to the user-defined anatomical regions^13^. Although these studies, e.g.,^10,12–14^, incorporated information from X-ray acquired at previous examination, their goal was to obtain better alignment between text and image modality, and their models were not able to generate radiology reports for disease progression-related findings.

### Image difference caption

The natural image difference captioning task, which aims to analyze the semantic differences between a pair of images^15,16^, is similar to the follow-up radiology report summary generation task.

Park et al. proposed a specialized attention mechanism to localize the changes between two images, the information of which was utilized in generating the corresponding spatially and temporally grounded difference caption^16^.

Shi et al. proposed a framework that generated difference caption by distinguishing semantic from viewpoint differences in image region by assessing feature similarity between different patches in two images^17^. Qiu et al. proposed a transformer-based framework for generating captions for multiple differences between an image pair by exploiting transformer blocks to capture image patch relationships and relevance between local regions^18^. Huang et al. proposed an instance-level feature extraction module that extracted fine-grained visual, semantic and positional features for better difference captioning^19^. Yao et al. proposed three contrastive learning-based pretraining tasks to promote fine-grained alignment between image differences and textual semantics^20^.

### Follow-up radiology report summary generation

The challenges of follow-up radiology report summary generation as compared to natural image difference captioning are: (1) the former is longer in text length and more diverse in text content than the latter, as it typically includes descriptions of the presence or absence of new and old diseases, as well as changes in disease severity; and (2) The differences between two X-rays are much more subtle, amidst significant variation in patient position and pose.

To the best of our knowledge, Hu et al. were the first to tackle the follow-up report summary generation task, and have assembled a MIMIC-Diff-VQA dataset with chest X-ray pairs and their corresponding follow-up report summary constructed from the MIMIC-CXR dataset^7^. They proposed a graph-based network to estimate the relationships between anatomical regions and diseases, namely spatial relationship for identifying abnormality locations, semantic relationship for capturing the interconnections among diseases, and implicit relationship for complementing the previous two relationships. However, they overlooked the importance of abnormality-related entities, and did not adequately emphasize the thereof in the generative process. In view of these shortcomings, in this study we devise a transformer-based framework to better model long-distance dependencies, and propose two specially designed mechanisms that aim at enhancing the prediction of medical lexicons for improving clinical accuracy and overall performance.

## Method

### Preliminaries

Given a pair of chest X-rays obtained at follow-up $\mathbf{I}^f$ and baseline examinations $\mathbf{I}^b$ from the same patient, our goal is to generate a summary of radiological findings with $N_s$ words ${\mathbf{s}=\{s_i\}^{N_s}_{i=1}}$ that describes disease progression at the time of follow-up examination. During model training, training dataset $\mathbb {D}$ consisting of X-ray pair $\{({\mathbf{I}^f}^{(i)},{\mathbf{I}^b}^{(i)})\}_{i\in \mathbb {D}}$ and the corresponding ground-truth follow-up radiology report summary $\{\mathbf{s}^{(i)}\}_{i\in \mathbb {D}}$ was used. Unless otherwise specified, the following formulation is concerned with a single sample for notional brevity.

$\mathbf{s}$ were tokenized $\mathbf{w}=\{w_i\}^{N_s}_{i=1}$, and were added with special token $\texttt {[BOS]}$ and appended with $\texttt {[EOS]}$ to respectively identify the beginning or ending of a sentence for facilitating the training process:

1 $$\begin{aligned} \bar{\mathbf{w}} = \left( \texttt {[BOS]}, w_1,\dots ,w_{N_s}, \texttt {[EOS]} \right) . \end{aligned}$$

Text encoding was subsequently performed via a word embedding layer $E_T$, which was initiated with training from the ground up:

2 $$\begin{aligned} \tilde{\mathbf{w}} = E_T(\bar{\mathbf{w}}). \end{aligned}$$

These word embedding were then augmented with sinusoidal positional encoding $x_i$ to obtain the input follow-up summary tokens :

3 $$\begin{aligned} \tilde{w}_i {:=}\tilde{w}_i + x_i \quad \forall i \in \{0,\ldots ,N_s+1\}. \end{aligned}$$

A pretrained image encoder $E_{I}$ was utilized to obtain visual features from $({\mathbf{I}^f},{\mathbf{I}^b})$:

4 $$\begin{aligned} \mathbf{V}^j = E_I(\mathbf{I}^j) \quad \forall j \in \{f,b\}, \end{aligned}$$

where $\mathbf{V}^b$ and $\mathbf{V}^f$ represent the grid features of the baseline and follow-up X-ray, respectively. These grid features were then flattened into a series of tokens, $\{\mathbf{v}_i^j \}_{i=1}^{N_v}$, and appended with a special token $\texttt {[} \texttt {Xray}^j \texttt {]}$, which captured the comprehensive representation of individual X-ray image:

5 $$\begin{aligned} \bar{\mathbf{V}}^j&= \left( \texttt {[} \texttt {Xray}^j \texttt {]}, \mathbf{v}_1^j,\dots , \mathbf{v}_{N_v}^j\right) \quad \forall j \in \{f,b\}. \end{aligned}$$

They were subsequently augmented with sinusoidal positional encoding $\mathbf{x}_i$ to obtain the input image tokens:

6 $$\begin{aligned} \bar{\mathbf{v}}^j_i {:=}\bar{\mathbf{v}}^j_i + \mathbf{x}_i \quad \forall i \in \{0,\ldots ,N_v\}, j \in \{f,b\}. \end{aligned}$$

Finally, additional type encoding were added to the input textual and image tokens to distinguish the source of these tokens.

**Fig. 2:**
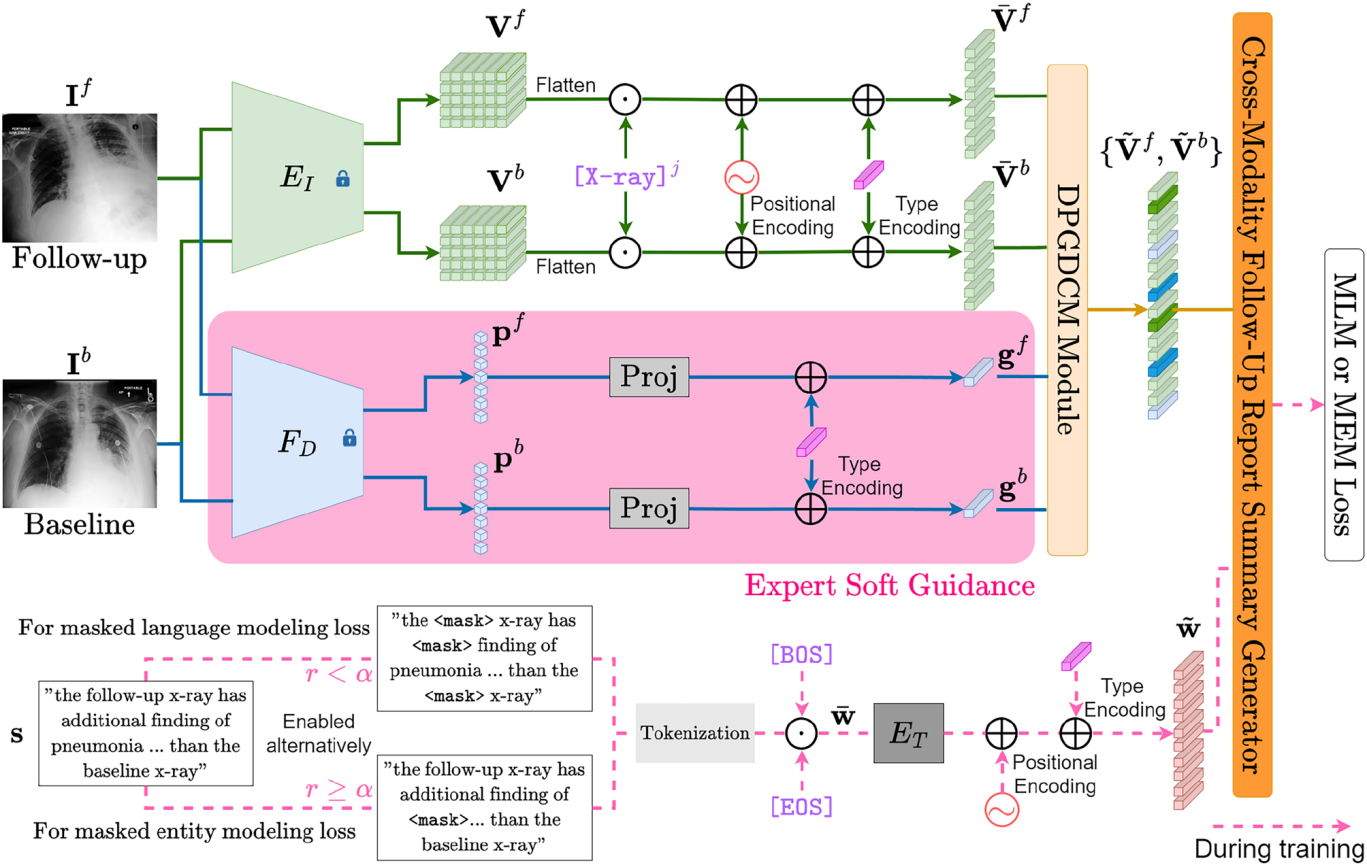
Illustration of our proposed framework, disease probability-enhanced (DPE) follow-up chest X-ray radiology report summary generation. The dotted arrows indicate the branches that were enabled only during model training. The lock symbol indicates module that were frozen. DPGDCM: disease probability-guided difference capture module; MLM: masked language modeling; MEM: masked entity modeling. $E_I$: image encoder; $F_D$: disease classifier; $E_T$: word embedding layer.

### Model architecture

The overall architecture of our proposed model, disease probability-enhanced (DPE) follow-up chest X-ray radiology report summary generation, is illustrated in Fig. 2. A pair of X-ray images were fed into a pretrained ResNet101^21^ to obtain image tokens, and a pretrained abnormality classifier to obtain the probability of the presence of abnormalities, dubbed soft guidance tokens. These tokens were fed into a transformer-based disease probability-guided difference capture module (DPGDCM) to obtain image difference representation. The image difference embeddings and follow-up summary tokens from report summary were input into the cross-modality follow-up report summary generator to generate a follow-up chest X-ray radiology report summary. This generative process was supervised by the masked language modeling (MLM) and masked entity modeling (MEM) losses.

#### Disease probability soft guidance

To bestow our model with clinical insight to enhance the prediction of abnormality-related entities, complementary guidance on the presence level of a finite number of chest X-ray abnormalities were obtained from a pretrained multi-class abnormality classifier $\text {F}_D$. Please refer to Section 4.3 for the list of abnormalities that the classifier could detect. Specifically, given a pair of chest X-ray images from follow-up and baseline examinations $(\mathbf{I}^f, \mathbf{I}^b)$, $\text {F}_D$ estimated the probability of the presence of abnormalities in each image:

7 $$\begin{aligned} \mathbf{p}^j&= \text {F}_D(\mathbf{I}^j) \quad \forall j \in \{f,b\}. \end{aligned}$$

These probabilistic outputs $\mathbf{p}^j$ thus served as complementary guidance on the presence of abnormalities. Rather than using a hard threshold to binarize these probabilities, they were directly mapped onto the same feature space of image tokens using a projection module $\text {Proj}(\cdot )$, which was a linear layer followed by a sigmoidal activation function:

8 $$\begin{aligned} \mathbf{g}^j&= \text {Proj} (\mathbf{p}^j) \quad \forall j \in \{f,b\}, \end{aligned}$$

where $\mathbf{g}^j$ represents the soft guidance token. Similar to the other input tokens, $\mathbf{g}^j$ were added with type encoding. These soft guidance tokens guided the DPGDCM in capturing a more abnormality-specific representation.

#### Disease probability-guided difference capture module

Disease probability-guided difference capture module, $\text {F}_{\text {diff}}$, was a two-layer transformer that was designed to estimate the representations of the difference in the image features of $(\mathbf{I}^f, \mathbf{I}^b)$. To better model long-distance dependencies in and merge all visual features, DPGDCM took all image tokens and guidance tokens as input:

9 $$\left\{ {\widetilde{{\mathbf{V}}}^{f} ,\;\widetilde{{\mathbf{V}}}^{b} } \right\} = {\text{F}}_{{{\text{diff}}}} \left( {\left\{ {\left( {\overline{{\mathbf{V}}} ^{f} ,{\mathbf{g}}^{f} } \right),\left( {\overline{{\mathbf{V}}} ^{b} ,{\mathbf{g}}^{b} } \right)} \right\}} \right).{\text{ }}$$

#### Cross-modality follow-up report summary generator

The cross-modality report generator $\text {F}_{\text {gen}}$ was a three-layer transformer that took image difference tokens and follow-up summary tokens as inputs:

10 $$\begin{aligned} (\hat{\mathbf{V}}^f,\hat{\mathbf{V}}^b,\hat{\mathbf{s}}) = \text {F}_{\text {gen}}(\tilde{\mathbf{V}}^f, \tilde{\mathbf{V}}^b, \tilde{\mathbf{w}}) \end{aligned}$$

#### Masked language modeling loss

Contrary to majority of vision-language pretraining works, uni-directional MLM loss was employed for the follow-up radiology report summary generation. The prediction of a masked word $s_i$ was based solely on its preceding words $s_{< i}$ and image tokens $(\hat{\mathbf{V}}^f, \hat{\mathbf{V}}^b)$. The same masking strategy as BERT^22^ was used, wherein 15% of the input text tokens were randomly selected. 80% of these randomly selected tokens were masked by the special token $\texttt {[MASK]}$, 10% substituted with words randomly chosen from vocabulary, and the remaining 10% left unchanged. To obtain the final predictions, the masked tokens were input into a linear classifier. Formally, the uni-directional MLM loss over $\mathbb {D}$ can be written as:

11 $${\mathcal{L}}_{{{\text{MLM}}}} = {\mathbb{E}}_{{({\mathbf{I}}^{f} ,{\mathbf{I}}^{b} ,{\mathbf{s}})\sim {\mathbb{D}}}} \left[ { - \log p\left( {s_{i} |h_{\Theta } \left( {{\mathbf{I}}^{f} ,{\mathbf{I}}^{b} ,s < i} \right)} \right)} \right]\quad \quad \forall i \in \left\{ {1, \ldots ,N_{s}+1 } \right\}.$$

where $h_{\Theta }$ is our proposed DPE model parameterized by $\Theta$.

#### Masked entity modeling loss

Masked entity modeling loss was proposed to direct our model’s attention toward abnormality-related words to improve their prediction. MEM was formulated similar to the MLM loss, but with the masking of the selected entity words:

12 $${\mathcal{L}}_{{{\text{MEM}}}} = {\mathbb{E}}_{{({\mathbf{I}}^{f} ,{\mathbf{I}}^{b} ,{\mathbf{s}})\sim {\mathbb{D}}}} \left[ { - \log p\left( {s_{i} |h_{\Theta } \left( {{\mathbf{I}}^{f} ,{\mathbf{I}}^{b} ,s < i} \right)} \right)} \right]\quad \quad \forall s_{i} \in {\mathcal{E}},$$

where $\mathscr {E}$ is the selected entity set. It is worth noting that in inference, no text inputs are required. The generation process is based on visual tokens and triggered by the input of $\left( \texttt {[BOS]}, \texttt {[MASK]}\right)$.

**Algorithm 1:**
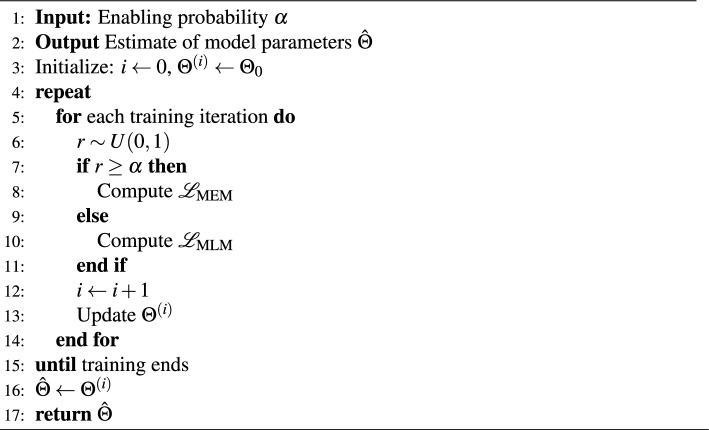
Alternating optimization strategy for MLM and MEM losses

**Table 2:** Comparison in the performance of our proposed disease probability-enhanced (DPE) follow-up chest X-ray radiology report summary generation against the state-of-the-arts.

Method	Natural language processing metric$^*$							Clinical metrics				
	B1	B2	B3	B4	M	R	C	Acc5	Acc14	F1-5	F1-14	Rad-F1$^\%$
$$\text {MCCFormers}$$ ^18^ $$^\dagger$$	21.4	19.0	17.0	15.3	31.9	34.0	0.0	–	–	–		
$$\text {IDCPCL}$$ ^20^ $$^{\dagger }$$	61.4	54.1	47.4	41.4	30.3	58.2	0.703	–	–	–		
EKAID^7^	62.8	55.3	49.1	43.4	33.9	57.7	1.027	$\text {68.2}^{\ddagger }$	$\text {81.0}^{\ddagger }$	$\underline{\text {50.1}^{\ddagger }}$	$\underline{\text {46.6}^{\ddagger }}$	$\underline{\text {31.6}^{\ddagger }}$
DPE-all	65.6	58.8	53.1	48.0	39.4	62.9	1.680	69.7	81.9	49.9	45.9	28.1
DPE-all14	66.2	59.8	54.3	49.2	40.0	64.9	1.762	71.3	82.8	49.6	46.4	27.1
DPE-all26	**69.0**	**62.6**	**57.2**	**52.4**	**42.2**	**69.2**	**2.118**	**76.5**	**85.2**	**58.5**	**55.9**	**36.7**
DPE-light	63.9	57.4	52.0	46.8	38.2	60.6	1.565	68.0	81.1	42.9	40.9	25.6

$\dagger$ Results were retrieved from ref.^7^. $\ddagger$ Results were obtained using the source codes of EKAID^7^. $^*$ B, M, R and C stand for BLEU, METEOR ROUGE-L and CIDEr respectively. $^\%$ Radgraph-F1. The best and second best performances were demonstrated in bold or underlined respectively

#### Ablation study

Ablation studies were performed to evaluate the effectiveness of our proposed disease probability soft guidance and MEM loss. Model that was trained using only the MLM loss, but without the disease probability soft guidance and the MEM loss, was considered as the baseline model, DPE-base. The variants of our proposed DPE models were: (1) DPE-mem: that trained using both the MLM and MEM losses; (2) DPE-esg: that trained using the MLM loss and disease probability soft guidance; (3) DPE-all: that trained using the MLM and MEM losses, and disease probability soft guidance.

### Training strategy

An alternating optimization strategy was adopted when training model with either of the MLM and MEM losses, the algorithm of which is illustrated in Algorithm 1. Specifically, for a given training iteration and hyperparameter $\alpha$, a random sample *r* was drawn from a uniform distribution between 0 and 1. If $r \ge \alpha$, the MEM loss was employed, and MLM loss otherwise. The optimization direction could thus be controlled by adjusting $\alpha$.

## Experiments

### Dataset and evaluation metrics

Our model was evaluated on the MIMIC-Diff-VQA^7^ dataset, which contains 164,324 pairs of X-rays and their corresponding follow-up radiology report summary. To evaluate the quality of the generated follow-up report summary, natural language processing (NLP) metrics, including BLEU^23^, METEOR^24^, ROUGE-L^25^ and CIDEr^26^, were used. Not only is it important to maintain linguistic quality, but also the clinical accuracy of the generated report summary. The accuracy of our model in correctly diagnosing a set of abnormalities was thus evaluated using Acc5, Acc14, F1-5, F1-14 and Radgraph-F1^1,2,27^. Acc5 is the micro-averaged accuracy over 5 most common abnormalities on chest X-ray, namely atelectasis, cardiomegaly, consolidation, edema, and pleural effusion. Acc14 is that over 14 abnormalities in CheXpert^28^, namely pneumonia, fracture, consolidation, enlarged cardiomediastinum, no finding, pleural other, cardiomegaly, pneumothorax, atelectasis, support devices, edema, pleural effusion, lung lesion and lung opacity^28^. Similar to Acc5 and Acc14, F1-5 and F1-14 are the F1-score over the aforementioned 5 or 14 abnormalities. Radgraph-F1 is the F1 score for assessing how well a model predicts the correct entities and their connections to ground truth. Note that instances that were labeled as negative, uncertain, or no mention were considered as negative finding.

**Table 3:** Ablation study on the effect of our proposed masked entity modeling loss and disease probability soft guidance on model performance.

Method	MEM$^{\dagger }$	ESG$^{\ddagger }$	Natural language processing metric							Clinical metric				
			B1	B2	B3	B4	M	R	C	Acc5	Acc14	F1-5	F1-14	Rad-F1
EKAID^7^			62.8	55.3	49.1	43.4	33.9	57.7	1.027	$\text {68.2}$	$\text {81.0}$	**50.1**	**46.6**	31.6
DPE-base			57.4	51.0	45.6	40.7	**41.6**	59.0	0.968	59.9	77.6	41.9	35.7	**32.7**
DPE-mem	$\checkmark$		61.4	54.7	49.2	44.1	40.5	61.6	1.421	65.9	79.1	43.3	42.3	29.5
DPE-esg		$\checkmark$	63.8	57.3	51.9	46.9	39.9	**63.6**	1.615	68.3	81.6	49.3	44.4	27.6
DPE-esg14		$\checkmark$	64.5	58.2	52.9	48.0	40.8	65.6	1.684	69.9	82.7	51.6	46.2	25.4
DPE-esg26		$\checkmark$	67.8	61.2	55.7	50.7	42.2	68.4	2.025	75.4	84.5	56.9	54.9	36.2
DPE-all	$\checkmark$	$\checkmark$	**65.6**	**58.8**	**53.1**	**48.0**	39.4	62.9	**1.680**	**69.7**	**81.9**	49.9	45.9	28.1
DPE-all14	$\checkmark$	$\checkmark$	66.2	59.8	54.3	49.2	40.0	64.9	1.762	71.3	82.8	49.6	46.4	27.1
DPE-all26	$\checkmark$	$\checkmark$	69.0	62.6	57.2	52.4	42.2	69.2	2.118	76.5	85.2	58.5	55.9	36.7

${\dagger }$ MEM: masked entity modeling loss. ${\ddagger }$ ESG: disease probability soft guidance

### Entity set

The entity set was comprised of chest X-ray abnormalities that occurred in both CheXpert and MIMIC-Diff-VQA datasets with more than 4% prevalence. These abnormalities included atelectasis, edema, pneumothorax, cardiomegaly, consolidation, fracture, lung opacity, pleural effusion, pneumonia, and cardiac silhouette. Note that enlargement of cardiac silhouette was also included in the entity set as it was frequently used in lieu of cardiomegaly in the MIMIC-Diff-VQA dataset.

### Implementation details

All experiments were conducted using either NVIDIA RTX-A6000 GPU or RTX-A100 GPU. A pretrained ResNet101^21^ was deployed as image encoder to extract image features with a size of $(7 \times 7 \times 2048)$, which were then flattened into a sequence of image tokens with a size of $(49 \times 2048)$. For all transformer-based modules, the hidden feature size was 512, the number of multi-heads was 8, and the number of layers were respectively 2 and 3 for the DPGDCM and cross-modality follow-up summary generator. The word embedding layer was learnt from scratch, and the feature dimension was set to 512. The hyperparameter $\alpha$ was set to 0.6, 0.2 and 0.7 for DPE-all, DPE-all14 and DPE-all26, respectively, and 0.8 for DPE-mem. The Adam optimizer with a learning rate of $3e^{-5}$ was used, and the maximum training iteration was set to $15e^4$. Early stopping was adopted to avoid overfitting. The hyperparameter $\beta$ was respectively set to 0.6 for the lightweight version of DPE-all (see Section 4.9 for more details).

Probabilistic class activation map (PCAM)^29^ was deployed as the disease classifier, and was dubbed Classifier-5. It utilized class activation map as weights to effectively aggregate visual information for enhancing classification performance, and was the top solution for CheXpert. It was trained on the CheXpert dataset and evaluated on the radiologist-labeled test set with mean AUC of 91.2 for classifying the presence of 5 abnormalities, namely atelectasis, cardiomegaly, consolidation, edema and pleural effusion^28^. Considering that the MIMIC-Diff-VQA dataset^7^ consists of abnormalities beyond those which PCAM could classify, we have also investigated whether the performance of our proposed framework would further improve if the abnormality classifier could provide guidance on more abnormalities. We therefore performed another two set of experiments using recently proposed classifiers, namely RaDialog^30^ and RAL^31^. RaDialog could provide guidance on all 14 abnormalities of the CheXpert dataset with a CheXbert^32^F1 score of 31.7^30^, denoted as Classifier-14. These abnormalities included pneumonia, fracture, consolidation, enlarged cardiomediastinum, no finding, pleural other, cardiomegaly, pneumothorax, atelectasis, support devices, edema, pleural effusion, lung lesion and lung opacity. For RAL, in addition to these CheXpert abnormalities, it could also provide guidance on 12 additional abnormalities, namely infiltration, nodule, mass, calcification of the aorta, emphysema, hernia, tortuous aorta, pleural thickening, emphysema, fibrosis, pneumomediastinum and pneumoperitoneum. The mean AUC of RAL was 83.7. It was trained on the CXR-LT dataset^33^, a long-tail extension of the CheXpert dataset by incorporating the aforementioned 12 tail-end findings.

To implement disease hard guidance, an additional threshold was selected to binarize each element of the probability outputs from the disease classifier $\mathbf{p}^j$, resulting in a presence indicator for each abnormality. This indicator vector was then passed through a projection module to produce the final guidance token, similar to the process of generating disease probability soft guidance in Eq. 8.

### Comparison with the state-of-the-arts

The performance of our proposed framework and state-of-the-arts (SOTAs) are shown in Table 2. Regardless of the classifier that was used for the disease probability soft guidance, our proposed framework outperformed the SOTAs in most metrics. In particular, the best performing variant was the DPE-all26, with improvements in NLP metrics ranging from 6.2 to 11.5, and clinical metrics range from 4.2 to 11.3, as compared to EKAID^7^. These results indicated that our framework was linguistically and clinically more accurate than SOTAs.

### Disease probability soft guidance for different number of abnormalities

As evident in Tables 2 and 3, the performance of our proposed framework improved with more abnormality guidance from abnormality classifier. The maximum performance gain in NLP metrics for DPE-all, DPE-all14 and DPE-all26, as compared to EKAID, were 0.65, 0.74 and 1.09, respectively. The improvement in Acc5 rose from 2.5 to 3.1 and 8.3, and that in Acc14 from 0.9 to 1.8 and 4.2. DPE-all26 achieved the best F1-5, F1-14 and Radgraph-F1 (7.4, 9.3 and 5.1 higher than EKAID, respectively). The improvement in performance with the use of Classifier-14 (DPE-all14) over Classifier-5 (DPE-all) in NLP metrics was up to 0.08, and that in Acc5, Acc14 and F1-14 were 1.6, 0.9 and 0.5, respectively. For the case of Classifier-26 (DPE-all26), improvement over Classifier-5 in NLP metrics was up to 0.44, and that in Acc5, Acc14, F1-5, F1-14 and Radgraph-F1 were 6.8, 3.3, 8.6, 9.3 and 8.6, respectively. Similar trend was also observed in the variant of DPE that only utilized disease probability soft guidance but not MEM loss. The improvement in performance with the use of Classifier-14 (DPE-esg14) over Classifier-5 (DPE-esg) in NLP metrics was up to 0.07, and that in Acc5, Acc14, F1-5 and F1-14 were 1.1, 1.6, 2.3 and 1.8, respectively. For the case of Classifier-26 (DPE-esg26), improvement over Classifier-5 in NLP metrics was up to 0.41, and that in Acc5, Acc14, F1-5, F1-14 and Radgraph-F1 were 7.1, 2.9, 7.6, 10.5 and 8.6, respectively. Taken together, these results indicated that the overall performance of our proposed framework improved with the availability of more disease probability soft guidance.

### Disease probability soft guidance versus disease hard guidance

Experiments were conducted to investigate the effect of disease probability soft guidance versus disease hard guidance on the performance of our proposed framework (DPE-all). As shown in Table 4, disease probability soft guidance permitted better performance than disease hard guidance. As much as 0.209 gain in NLP metric, and 4.1 in Acc5, 1.5 in Acc14, 2.3 in F1-5, 2.5 in F1-14 and 2.4 in Radgraph-F1 were observed. Note that the gain in performance by disease probability soft guidance over disease hard guidance changed with the threshold for hard guidance. These results affirmed that disease probability soft guidance was more effective and robust, as it eliminated the need for careful hyperparameter tuning.

### Masked entity modeling loss versus masked language Modeling Loss

**Fig. 3:**
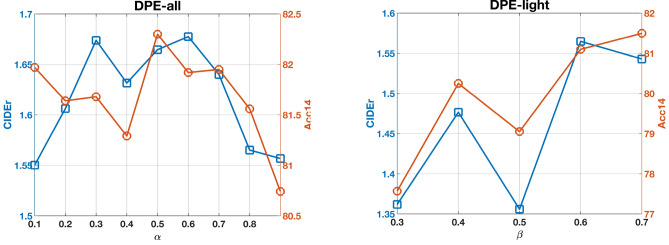
(**a**) The effect of the alternating optimization strategy for MLM and MEM losses on the performance of DPE-all. $\alpha$ is the enabling probability that controlled which of the two losses was used at a given training iteration. (**b**) Hyperparameter sensitivity study of DPE-light for different probability $\beta$ on enabling disease probability soft guidance.

Whether the MLM or MEM loss was used in a given model training iteration depended on the enabling probability $\alpha$. The effect of this alternating optimization strategy on the performance of our proposed framework (DPE-all) was thus investigated (see Fig. 3a). In either of the scenario where one loss was dominated by the other, the linguistic quality (CIDEr) of the generated report summary was largely similar. The peak CIDEr occurred when $\alpha =0.6$. The clinical accuracy (Acc14) of the generated report summary when MEM loss was predominantly used, i.e., $\alpha =0.1$, was higher than that when MLM loss was predominantly used, i.e., $\alpha =0.9$. The peak Acc14 occurred when $\alpha =0.5$. These results affirmed that the use of our proposed MEM loss permitted the generation of report summary with higher clinical accuracy.

**Table 4:** The effect of disease probability soft guidance versus disease hard guidance on the performance of our proposed DPE-all.

Hard threshold	Natural language processing metric							Clinical metric				
	B1	B2	B3	B4	M	R	C	Acc5	Acc14	F1-5	F1-14	Rad-F1
0.5	63.9	57.0	51.3	46.1	38.0	59.9	1.525	67.0	80.7	47.6	43.4	25.7
0.6	63.7	56.8	51.1	45.9	38.4	60.3	1.511	66.5	80.7	47.9	43.6	26.0
0.7	63.6	56.8	51.0	45.8	38.6	60.6	1.506	66.3	80.8	48.1	43.7	26.3
0.8	63.3	56.5	50.6	45.5	39.0	60.8	1.479	65.8	80.6	48.3	43.7	26.8
0.9	62.9	56.1	50.3	45.2	39.3	60.9	1.471	65.6	80.4	47.9	43.8	27.3
Soft	**65.6**	**58.8**	**53.1**	**48.0**	**39.4**	**62.9**	**1.680**	**69.7**	**81.9**	**49.9**	**45.9**	**28.1**

### Ablation study

The efficacy of our proposed disease probability soft guidance and MEM loss were illustrated in Table 3. Compared to the baseline model (DPE-base), our proposed mechanisms and combination of the thereof permitted notable gain in performance in NLP and clinical metrics, with the exception of Radgraph-F1. For DPE-mem, improvement in NLP metric was up to 0.45, and that in Acc5, Acc14, F1-5 and F1-14 were 9.0, 1.5, 1.4 and 6.6, respectively. For DPE-esg, improvement in NLP metric was up to 0.67, and that in Acc5, Acc14, F1-5 and F1-14 were 8.4, 4.0, 7.4 and 8.7, respectively. For DPE-all, improvement in NLP metric was up to 0.71, and that in Acc5, Acc14, F1-5 and F1-14 were 9.8 and 4.3, 8.0 and 10.2, respectively. Of note is that the trend for Radgraph-F1 was largely reversed. Nevertheless, the use of Classifer-26 (i.e., DPE-esg26 and DPE-all26) permitted our proposed framework to outperform DPE-base and EKAID by a large margin.

Taken together, although both of our proposed mechanisms alone permitted better performance in NLP and clinical metrics, with the exception of Radgraph-F1, over baseline, only DPE-esg and DPE-all were able to outperform SOTAs. The same trends were also observed for variants that utilized Classifier-14 and Classifier-26. In other words, the benefit of MEM loss in the generation process of follow-up radiology report summary would only manifest when disease probability soft guidance was utilized. Taken together, these results suggested that maximum performance gain over SOTAs required both of our proposed disease probability soft guidance and MEM loss.

**Algorithm 2:**
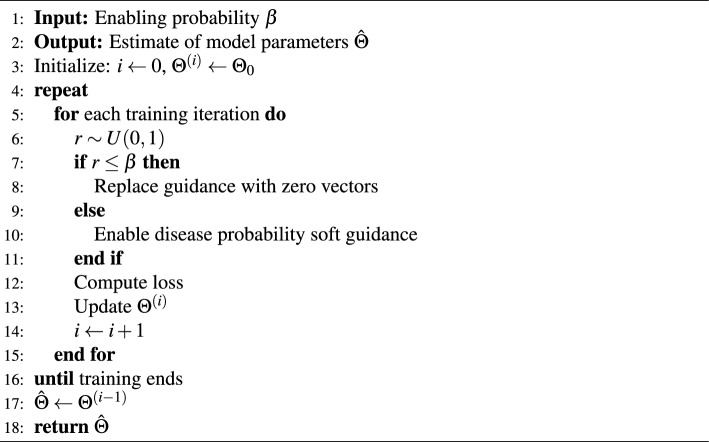
Random dropout training strategy

**Fig. 4:**
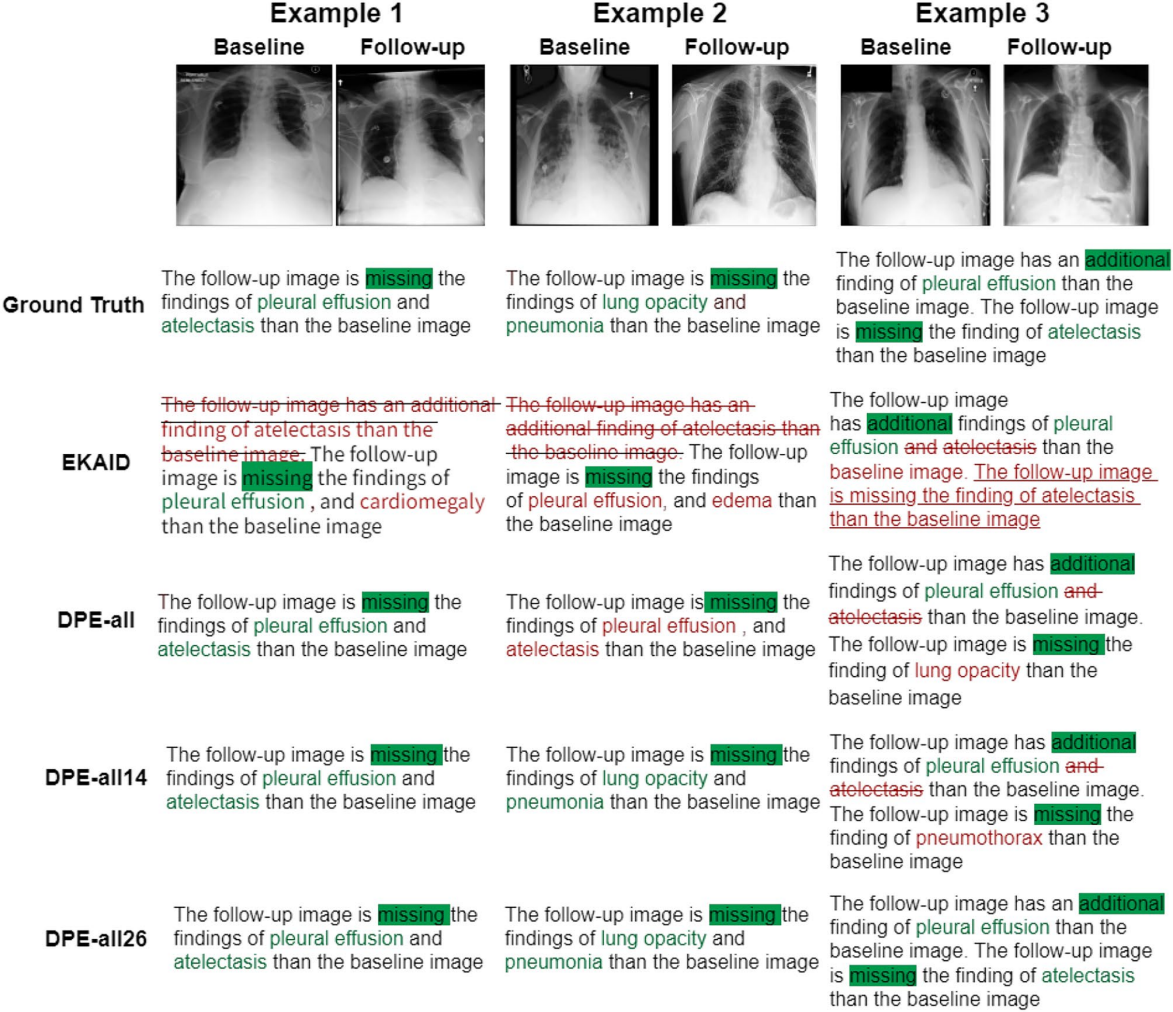
Qualitative evaluation of three representative cases. The green background and font color represent correct predictions of important vocabulary or medical terms. The red underline indicates that the finding that was missed by the model, while the red strikethrough represents hallucination. The red font color indicates incorrect predictions.

### Lightweight framework

Considering the importance of saving computation resources and achieving faster inference, we investigated whether model performance could be maintained by streamlining version of DPE-all (denoted as DPE-light) such that disease probability soft guidance was utilized only during the training phase, but not model inference. To achieve this objective, we proposed a random dropout training strategy, which is described in Algorithm 2. Specifically, an enabling probability $\beta$ was assigned to the disease probability soft guidance. During a random training iteration, a sample *r* is drawn from a uniform distribution between 0 and 1. If $r<\beta$, the guidance from the abnormality classifier was replaced with zero vectors of the same dimension. Otherwise, the guidance was enabled and put into the projection module. The results of DPE-light are demonstrated in Table 2, and the results of the hyperparameter sensitivity study of $\beta$ are illustrated in Fig. 3b.

Notably, DPE-light surpasses state-of-the-art methods on NLP metrics by a considerable margin, achieving gains of 1.1%, 2.1%, 2.9%, and 3.4% in BLEU, along with enhancements of 4.3% and 2.9% in METEOR and ROUGE-L, respectively. Furthermore, there is a notable 0.538 improvement in CIDEr, representing a 52.39% increase. However, for clinical metrics, DPE-light was not different from EKAID, whereby ACC5 is a bit lower (0.2%) and ACC14 slightly better (0.1%). It is worth mentioning that DPE-light exhibits superior performance compared to DPE-mem and comparable performance to DPE-esg, indicating that even without guidance during inference, the guidance during training significantly enhances the performance of DPE-base and DPE-mem.

As shown in Fig. 3b, the CIDEr of DPE-light was consistently better than EKAID (at 1.35) across all $\beta$, and peaked at 1.565 at $\beta =0.6$. For Acc14, DPE-light peaked at 81.1, 0.1% higher than EKAID, at $\beta =0.7$. Taken together, DPE-light was competitive with but could not exceed EKAID.

### Radiologist evaluation

A comparative analysis was performed to evaluate the extent of agreement in follow-up report summary between radiologists and follow-up report summary generation models. A clinical (W.H.C.; with over 10 years of board certified experience) and an academic (T.Y.S.; with over 10 years of experience) radiologists performed the evaluation. Each radiologist was given the same 25 pairs of follow-up and baseline X-ray images randomly selected from the MIMIC-Diff-VQA dataset, and the corresponding generated report summary from EKAID, DPE-light and DPE-all26. An 5-point agreement score (RADPEER, developed by the American College of Radiology) was used to assess discrepancies and concordances: 1, clinically important discrepancy likely to affect patient management; 2, discrepancy in interpretation but unlikely to affect patient management; 3, minor discrepancy with clinical impact; 4, reasonable disagreement with no clinical impact; and 5, complete agreement between interpretations. All combining the scores from the two radiologists, the median score (inter-quartile range) for EKAID, DPE-light and DPE-all26 were 2 (1–3), 3 (2–4.75) and 3 (2–4), respectively. These results suggested that our proposed framework also outperformed SOTA from the perspective of radiologists.

### Qualitative evaluation

Qualitative evaluations of the generation of follow-up report summary were performed (see Fig. 4). For Example 1, all models could correctly generate the *absence* of *pleaural effusion*. EKAID hallucinated on *new findings of atelectasis*, and wrongly generated the *absence of cardiomegaly*. Our proposed framework could correctly report the *absence of atelectasis*. For Example 2, EKAID hallucinated on the *new findings of atelectasis*. The *absence of lung opacity and pneumonia* were missed by DPE-all and EKAID, and were correctly generated by DPE-all14 and DPE-all26. For Example 3, EKAID failed to report the *missing finding of atelectasis*, and wrongly treated it as a new finding. Whilst all models could correctly generate the *new findings of pleural effusion*, DPE-all and DPE-all14 hallucinated on the *new findings of atelectasis*. DPE-all and DPE-all14 respectively wrongly generated the *missing finding of lung opacity and pneumothorax*, DPE-all26 could correctly generate the *absence of atelectasis*.

## Limitations

There are several limitations that may hinder the performance of our proposed framework. Our proposed disease probability soft guidance lacks disease severity and position information, which could respectively be provided by a level classifier and a segmentation or detection model to potentially further improve model performance. Second, our proposed framework could potentially be further improved when prior information from baseline radiology report is incorporated. This is grounded on the evidence from the study by Bannur et al. who showed that the context from baseline report played a key role in the quality of the generated radiology report for the follow-up X-ray as it summarized the baseline X-ray image and provided a richer signal for model training^12^. In our future work, we plan to enhance our current model by integrating baseline reports to improve the quality of generation, while ensuring it retains the capability to generate follow-up reports even in the absence of baseline information. Finally, the performance may be enhanced once we adapt our framework to higher resolution inputs, especially for diseases that require detailed visual information, as evidenced by recent works^34^.

## Conclusion

We have proposed a transformer-based framework to address the clinically critical, and yet largely understudied, task of follow-up chest X-ray radiology report summary generation. Two mechanisms, namely disease probability soft guidance and masked entity modeling loss, were proposed to bestow clinical insight on abnormality recognition for improving the overall model performance. Extensive experiments demonstrated that the performance of our model exceeded the state-of-the-arts.

## Data Availability

We used the open-sourced MIMIC-Diff-VQA dataset, which was sampled from MIMIC-CXR and contained 164,324 pairs of X-rays and their corresponding follow-up radiology report summary.
